# In vitro and in vivo anti-inflammatory and anticoagulant activities of *Myrciaria plinioides* D. Legrand ethanol leaf extract

**DOI:** 10.1007/s10787-022-00924-0

**Published:** 2022-02-14

**Authors:** Diorge Jônatas Marmitt, Shanna Bitencourt, Chistiane Oliveira Coura, Markus Berger, Dalana Faleiro, Débora Mara Kich, Bruna Caye, Sheila Mariele Immich, Annyta Fernandes Frota, Walter O. Beys-da-Silva, Jorge Almeida Guimarães, Norma Maria Barros Benevides, Stefan Laufer, Márcia Inês Goettert

**Affiliations:** 1 Laboratório de Cultura de Células, Programa de Pós-Graduação em Biotecnologia, Universidade do Vale do Taquari-Univates, Lajeado, Brazil; 2 Departamento de Bioquímica e Biologia Molecular, Universidade Federal do Ceará (UFC), Fortaleza, Brazil; 3 Laboratório de Bioquímica Farmacológica, Centro de Pesquisa Experimental, Hospital de Clínicas de Porto Alegre, Universidade Federal do Rio Grande do Sul (CPE-HCPA/UFRGS), Porto Alegre, Brazil; 4 Programa de Pós-Graduação em Biologia Celular e Molecular, Centro de Biotecnologia, Universidade Federal do Rio Grande do Sul, Porto Alegre, Brazil; 5 Faculdade de Farmácia, Universidade Federal do Rio Grande do Sul, Porto Alegre, Brazil; 6 Department of Pharmaceutical and Medicinal Chemistry, Institute of Pharmacy, University of Tübingen, Tübingen, Germany

**Keywords:** *Myrciaria plinioides*, Anti-inflammatory activity, p38 signaling pathway, Rat model, Anticoagulant, Extrinsic pathway

## Abstract

*Myrciaria plinioides* D. Legrand (Myrtaceae) is a native plant of Southern Brazil, which have potential in the food industry due to its edible fruits. Many plants belonging to this genus have been used for a variety of illnesses, including inflammatory disorders due to antioxidant properties. However, therapeutic uses of *M. plinioides* have been poorly studied. The aim of study was to assess the anti-inflammatory and anticoagulant activities of the ethanol leaf extract of *M. plinioides*. In *M. plinioides* extract-treated RAW 264.7 cells, assessments of cell viability, TNF-α release and p38 MAPK pathway-dependent protein expression were detected. In addition, rat paw edema models were used to analyze the anti-inflammatory effect of the extract. Macrophages cell line treated with *M. plinioides* extract showed a slight decrease in cell viability. In LPS-stimulated macrophages treated with different concentrations of the extract for 24 h, TNF-α release was inhibited, while modulation of p38 signaling pathway and inhibition of NF-κB p65 protein expression were dose-dependent. In rats, the extract inhibited the formation of paw edema, while an inhibitory effect on trypsin-like enzymes derived from mast cells was seen. Furthermore, the extract presented anticoagulant activity via extrinsic pathway, being able to block specifically factor Xa and thrombin. The study suggests that extract possess potent anti-inflammatory and anticoagulant effects. *M. plinioides* present great biological potential as a source for the development of anti-inflammatory and anticoagulant drugs. Additional studies can be proposed to better elucidate the mechanism by which *M. plinioides* exerts its effects.

## Introduction

Inflammatory response is a complex phenomenon of vascular tissues triggered by harmful stimuli, which can be manifested through a variety of mechanisms leading to activation of immune cells, release of cytokines and vasoactive mediators, and activation of coagulation factors, including tissue factor (TF) and other procoagulant factors (Newton and Dixit 2012). Several signaling pathways are activated during inflammation to modulate proliferation, differentiation and cell death in an effort to sustain tissue homeostasis and local blood flow (Li et al. 2017).

Mainly macrophages, mast cells and neutrophils have a key role during inflammatory response as they can release a variety of mediators including vasoactive amines, proteolytic enzymes (e.g., trypsin, tryptase, chymase and cathepsins), proinflammatory cytokines (e.g., tumor necrosis factor-alpha, TNF-α) and also can trigger proteolytic cascades, such as the kallikrein–kinin system and blood coagulation (Bender et al. 2017). In fact, in situations of inflammation, such as those triggered by the pathogen molecule lipopolysaccharide (LPS), cytokines such as TNF-α, interleukin-1β (IL-1β) or IL-6 are able to induce TF expression on endothelial cells and monocytes, leading it to a procoagulant profile (Pawlinski et al. 2004). Once expressed in cell membrane of endothelial cells and/or monocytes, TF can bind and activate FVII generating FXa and active thrombin, which can, in turn, bind to protease activated receptors (PAR) on platelets and other pro-inflammatory cells amplifying the inflammatory signal (Landis 2007). This mechanism represents the link between inflammation and coagulation and the discovery of new molecules able to modulate key enzymes or cytokines in this process is very interesting to treat several vascular disorders. In addition, modulation of specific signaling pathways, such as mitogen-activated protein kinases (MAPKs) and nuclear factor-kappa B (NF-κB), which can be induced by oxidative stress, are important targets in the discovery of new drugs for the treatment of a wide range of inflammatory disorders (Laufer et al. 2002).

Natural products, including compounds derived from plants, have been used for the development of new drugs, one of the main examples being acetylsalicylic acid (Aspirin), extracted from the bark of the plant *Salix alba* L. Antioxidant substances based on natural compounds play a preventive role in protecting against the generation of free radicals and, therefore, are one of the most valuable therapeutic agents for reducing the various associated inflammatory diseases (Shara and Stohs 2015). For example, in addition to having antioxidant activities, flavonoids and phenolic compounds also play an effective role as anti-inflammatory factors, blocking two main signaling pathways, such as NF-κB and MAPKs, which have the main role in the production of several mediators proinflammatory (Arulselvan et al. 2016).

In this sense, several studies reported that members of Myrtaceae family present medicinal properties and are widely used in folk medicine (Borges et al. 2014). However, there are few studies regarding the biological effects of species of the genus *Myrciaria*, popularly known as ‘jabuticabeiras’, with potential in the food industry because of its edible flavored fruits and the presence of flavonoids and phenolic compounds (Wang et al. 2014). In addition to their use as food, many of the fruits, seeds and leaves of plants belonging to this genus have been used in traditional medicine for a variety of illnesses and conditions, including inflammatory disorders and diseases with oxidative components (Pietrovski et al. 2008). *Myrciaria plinioides* D. Legrand is a species belonging to this genus; however, the therapeutic uses and potential values of this plant have been poorly studied (Marmitt et al. 2019). Therefore, this work aimed to evaluate the in vitro and in vivo effects of *M. plinioides* ethanol leaf extract on the inflammatory response and hemostasis.

## Materials and methods

### Reagents

Reagents were purchased from Sigma-Aldrich (St. Louis, MO, USA) unless stated otherwise. Antibodies for p38α MAPK: sc-535; p-p38α MAPK: sc-166182; NF-κB: sc-56735; β-actin: sc-81178, were purchased from Santa Cruz Biotechnology (Santa Cruz, CA, USA). Opti-4CN substrate kit was acquired from Bio-Rad Laboratories (Hercules, CA, USA). Synthetic substrates for thrombin (S-2238™, H-D-Phe-Pip-Arg-pNa), factor XIa (S-2366, pyroGlu-Pro-Arg-pNa), plasma kallikrein and factor XIIa (S-2302™, H-D-Pro-Phe-Arg-pNA), factor Xa (S-2222™, Bz-Ile-Glu-Gly-Arg-pNa) and factor VIIa (SCP-0248, MeSO2-Cha-Abu-Arg-pNA)) were purchased from Chromogenix (Milan, Italy). Aprotinin (Trasylol^®^) was purchased from Bayer (São Paulo, Brazil). Ellagic acid, rabbit brain thromboplastin, phospholipids were purchased from Wiener Lab (Rosario, Argentina).

### Plant material, cell line, maintenance and viability

A voucher specimen was deposited at the Herbarium of Univates (1066). We stated that the plant name has been checked with http://www.theplantlist.org. RAW 264.7 murine macrophage cell line was obtained from the Rio de Janeiro Cell Bank (BCRJ, # 0212), Brazil. Cells were cultured in DMEM medium supplemented with 10% fetal bovine serum and 1% antibiotics. Cells were incubated at 37˚C in a humidified atmosphere containing 5% CO_2_.

The assessment of cell viability was performed using two methods: Alamar Blue™ and MTT, according to manufacturer’s instruction. Cells were seeded in 96-well microplates and challenged with increasing concentrations of ethanol extract (50, 100 and 200 µg/mL). After 48 h incubation (Alamar Blue) or 24 h (MTT), the absorbance was read at 540 nm. The extract doses were in accordance to Marmitt et al. 2019.

### LPS-induced TNF-α release inhibition

RAW 264.7 cells with or without 1 h extract pre-treatment (25–200 µg/mL) were stimulated with LPS (1 µg/mL) for 24 h. TNF-α concentration in the supernatants were quantified by ELISA (Invitrogen) according to manufacturer’s instruction. The results were presented as picograms per milliliter (pg/mL).

### Protein extraction and western blot analysis

RAW 264.7 cells were incubated in the presence of different concentrations of the extract (25, 50 and 100 µg/mL) and 1 h later, LPS (1 µg/mL) was added. After 24 h of incubation, cells were washed twice with ice-cold PBS and incubated for 30 min on ice with lysis buffer (150 mM NaCl, 1 mM EDTA, 50 mM Tris pH 7.4, 0.1% Triton ×100, and 0.1% SDS) supplemented with protease and phosphatase inhibitors. The protein concentration was measured using Lowry method. Fifty micrograms of protein were separated on 10% Tris–glycine SDS–Polyacrylamide gels and transferred onto nitrocellulose membranes using a semidry blotting apparatus. For western blot analysis, the membranes were blocked in 10% milk in TBS-Tween. After overnight incubation with primary antibodies (1/500) (p38α, p-p38α MAPK, NF-κB and β-actin) and 2 h incubation with horseradish peroxidase conjugated secondary antibodies (1/5000), proteins were visualized with the Opti-4CN colorimetric detection kit. Protein band intensities were quantified using the program ImageJ 1.48v.

### Animal models of inflammation

The research was conducted in accordance with internationally accepted principles for laboratory animal use and care. All procedures with animals were approved by the Ethical Committee on Animal Research of the Federal University of Ceará, Brazil (CEPA # 71/2012). Male Wistar rats (120–160 g) were used in the study. Rats were kept in the Animal Care Facility with a 12 h light–dark cycle at constant temperature, with free access to water and chow during the study period. The paw edema models were assayed according to Coura et al. (2015).

### Carrageenan-induced paw oedema assay

Animals (*n* = 6/group) were given increasing doses (50, 100 and 200 mg/kg BW; for each 100 g of BW it was used 100 µL of DMSO) of *M. plinioides* extract subcutaneously (sc) into the back of each animal. Saline was used as internal control of the experiment, while the glucocorticoid dexamethasone (1 mg/kg, sc) was used as reference drug. Paw edema was induced by intraplantar injection of 100 μL carrageenan solution (700 μg/paw) into the back of the right hind paw after 1 h of drug administration. The volume of the right paw was measured using a plethysmometer and the results were presented as variations in the paw volume (mL), which were calculated relative to the basal volume (time 0).

### Dextran-induced paw oedema assay

Sterile saline (0.9%, 100 μL) or *M. plinioides* ethanol extract (50, 100 and 200 mg/kg BW) were sc administered into the back of the rats 1 h before dextran (500 μg/paw)-induced oedema by injection into the right hind paw. Variations in the paw volume (mL) were calculated relative to the basal volume (time 0).

### Inhibition of mast cell proteolytic activity

A suspension enriched in mast cells was obtained by peritoneal lavage of rats pre-stimulated with carrageenan. Mast cells were purified after centrifugation in 70% Percoll gradient solution as described by Kovarova (2013). The mast cell suspension was macerated in PBS, centrifuged at 3000 g for 15 min and the protein extract obtained was 1:5 diluted in the same buffer and incubated with different concentrations of the extract (0–600 μg/mL). The residual activity of the trypsin-like enzymes released from mast cells was measured by the addition of a synthetic chromogenic substrate (BAPNA-L-benzoyl-arg-pNa). Kinetics of p-nitroaniline formation were monitored at 405 nm in 14 s intervals for 30 min. Results were expressed as the initial velocity of trypsin-like enzymes (mOD/min).

### Effects of M. plinioides extract on hemostatic system

#### Coagulation assays

Human venous blood or rabbit, rat and bovine blood were collected from healthy subjects in 1:10 (v/v) 3.8% trisodium citrate and centrifuged at 1500×*g* for 10 min to obtain plasma. The anticoagulant activity of *M. plinioides* ethanol extract (0–1200 µg/mL) was verified by determination of the following coagulation parameters in plasma: recalcification time (RT), activated partial thromboplastin time (aPTT) and prothrombin time (PT). Evaluation of these parameters was performed using commercial kits following manufacturer’s instructions (Wiener Lab, Rosario, Argentina). The kinetic of all assays were conducted using a 96-well microplate spectrophotometer (SpectraMax 190, Molecular Devices Co., Sunnyvale, CA, USA) at 650 nm equipped with temperature control and shaking systems as previously described (Ribeiro 1995). Results were expressed as coagulation time (measured in seconds).

#### Amidolytic activity of intrinsic pathway factors (FXIIa, FXIa and kallikrein) in plasma

The inhibitory capacity of *M. plinioides* ethanol extract on the amidolytic activity of factors XIIa, XIa and kallikrein was measured directly in the plasma using chromogenic substrates and specific inhibitors for each factor. Human plasma was treated with 2 mM acetic acid and diluted 1:10 in 20 mM Tris–HCl buffer, pH 7.5. Subsequently, 50 μL of the diluted plasma was incubated with *M. plinioides* extract (90 μg/mL) for 10 min at 37 °C and then the intrinsic factors were activated by the addition of 20 μL of ellagic acid. The residual XIa, XIIa and kallikrein activities produced during reactions in the presence or absence of the extracts were determined in three different experiments: (*i*) The activity of factor XIa was measured by the addition of 2 mM of the substrate S-2366 (pyroGlu-Pro-Arg-pNA) in the presence of aprotinin (50 μM) and a Bowman–Birk trypsin inhibitor (SBTI, 100 nM); (*ii*) the activity of factor XIIa formed was measured by the addition of 2 mM of substrate S-2302 (H–D-Pro-Phe-Arg-pNA) in the presence of aprotinin (50 μM); and (*iii*) the activity of the kallikrein formed was measured by adding S-2302 (2 mM) in the presence of SBTI (100 nM). In all cases the kinetics of *p*-nitroaniline formation were monitored (405 nm) at a time interval of 14 s during a total time of 30 min on the microplate reader spectrophotometer (SpectraMAX 190, Molecular Devices, Sunnyvale, CA, USA). Results are expressed as percentage of amidolytic activity.

#### Amidolytic activity of extrinsic pathway factors (FXa and thrombin) in plasma

The inhibitory capacity of *M. plinioides* ethanol extract on the amidolytic activity of FXa and thrombin was measured directly in plasma using specific chromogenic substrates and human plasma deficient for each factor. To measure the inhibitory capacity of the extract on factor Xa, prothrombin-deficient human plasma (50 μL) was activated with tissue factor (0.17 mg/mL) in the presence of CaCl_2_ (7 mM) and incubated for 10 min at 37 °C with *M. plinioides* extract (90 μg/mL). The amidolytic activity of factor Xa produced during incubation time was measured by addition of 2 mM of substrate S-2222 (Bz-Ile-Glu (γ-OR) -Gly-Arg-pNA). To measure the inhibitory capacity of the extract over thrombin, factor X-deficient human plasma was diluted 1:10 in 20 mM Tris–HCl buffer, pH 7.5. Then, 50 μL of the diluted plasma was activated with *Oxyurannus scutellatus* snake venom (10 μg/ mL) in the presence of CaCl_2_ (7 mM) and incubated for 10 min at 37 °C with the *M. plinioides* extract (90 μg/mL). The amidolytic activity of the thrombin produced during the incubation time was measured by adding 2 mM of the substrate S-2238 (H-D-Phe-Pip-Arg-pNA). In both cases the kinetics of *p*-nitroaniline formation were monitored (405 nm) at a time interval of 14 s during a total time of 30 min on the microplate reader spectrophotometer (SpectraMAX 190, Molecular Devices, Sunnyvale, CA, USA). Results were expressed as a percentage of amidolytic activity.

### FVa activity in plasma

The inhibitory capacity of the *M. plinioides* extract on the activity of FVa was evaluated by the prolongation of PT in FV deficient plasma. For this purpose, the extract (90 μg/mL) were incubated with human normal plasma (50 μL) or factor V deficient plasma (50 μL) for 10 min at 37 °C. The extrinsic pathway was activated by the addition of rabbit brain thromboplastin (100 μL) in the presence of CaCl_2_ (10 mM) and phospholipids. The experiments were carried out as previously described, with reaction kinetics being monitored at 650 nm for 5 min with readings at time intervals of 5 s. The results are expressed as coagulation time in seconds and represent the mean ± standard error of three independent experiments. The absence PT prolongation in FV-deficient plasma indicates the presence of a specific inhibitor of factor Va.

### FVIIa activity in plasma

The amidolytic activity of FVIIa was measured directly in the plasma using its specific chromogenic substrate. For this purpose, factor × deficient human plasma (50 μL) was activated with tissue factor (0.17 mg/mL) in the presence of CaCl_2_ (7 mM) and incubated for 10 min at 37 °C with *M. plinioides* extract (90 μg/mL). The amidolytic activity of FVIIa produced during incubation time was measured by the addition of 2 mM SCP-0248 substrate (MeSO2-Cha-Abu-Arg-pNA). The kinetics of *p*-nitroaniline formation were monitored (405 nm) at a time interval of 14 s during a total time of 30 min on the microplate reader spectrophotometer (SpectraMAX 190, Molecular Devices, Sunnyvale, CA, USA).

## FXa and thrombin inhibition

The ability of *M. plinioides* extract to block FXa and thrombin activities was measured by prothrombin activation and thrombin-induced fibrinogen clotting, respectively. Briefly, purified FXa (3.4 μg/mL) was incubated with *M. plinioides* extract (0–600 μg/mL) for 10 min at 37 °C. Purified prothrombin (34 μg/mL) was then added in the presence of CaCl_2_ (7 mM) and incubation was maintained for a further 20 min. Thrombin formed during the activation process was monitored by the addition of fibrinogen (2 mg/ml, final concentration) at 650 nm. For thrombin-induced fibrinogen clotting assay purified thrombin (2 μg/mL) was incubated with *M. plinioides* extract at different concentrations (0–900 μg/mL) for 10 min at 37 °C. Fibrin formation was monitored by the addition of fibrinogen (2 mg/ml) at 650 nm. Results were expressed as percentage of FXa and thrombin inhibition. IC_50_ values were determined by exponential nonlinear regression analysis.

### Statistical analyses

All experiments were performed at least in triplicate and data were presented as mean ± SEM. Data were analyzed and graphed using Prism 5.0 (Graphpad Software, Inc). Statistical significance was determined using one-way ANOVA with Dunnett’s or Tukey’s correction for multiple comparisons or unpaired two-sided Student’s *t* test. In vivo experiments were analyzed using two-way ANOVA with Dunnett’s correction for repeated measures. A *p* value < 0.05 was considered statistically significant.

## Results

### Anti-inflammatory effect of M. plinioides extract on RAW 264.7 cells

**Fig. 1:**
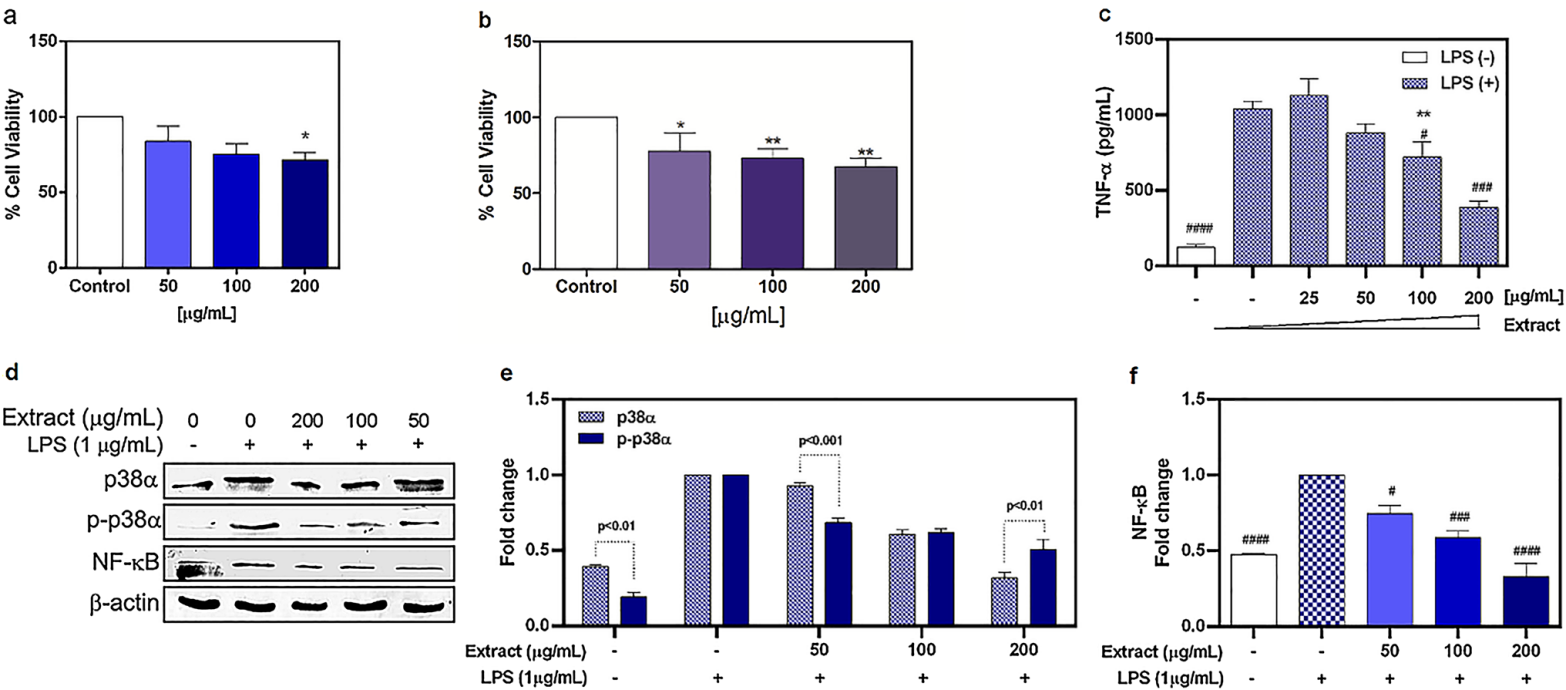
Effects of *M. plinioides* ethanol extract on RAW 267.4 cells. **a** Cells were challenged with different concentrations of the extract for 48 h and cell viability was assessed using Alamar blue assay. **b** Macrophages treated with extract were evaluated by the MTT method. **c** Cells were 30 min pre-incubated with the extract and then challenged with LPS for 24 h. Then, TNF-α release was quantified by ELISA. DMSO was used as control. **d** Representative western blot analysis of p38α, p-p38α and NF-kB protein expressions after 24 h treatment. Cells were treated with increasing concentrations of ethanol extract and LPS was added after 1 h. β-actin was used as a loading control. Relative quantifications of **e** p38α and p-p38α, and **f** NF-kB proteins, respectively. Mean values ± SEM are shown (*n* = 3). *p < 0.05, **p < 0.01 compared with control; ^#^*p* < 0.05, ^##^*p* < 0.01, ^###^*p* < 0.001, ^####^*p* < 0.0001 compared with LPS. Control groups, cells without any treatment

To assess the potential in vitro anti-inflammatory effects of *M. plinioides* ethanol extract, we used the murine macrophage RAW 264.7 cell line. First, we evaluated the viability of cells challenged with increasing concentrations of the extract in two methods. As shown in Fig. 1a, b, a slight decrease (*p* < 0.05) in viability was observed only in cells treated with the highest concentration (200 μg/mL) of the extract in both cell viability assays. After, we investigated the effect of the extract on TNF-α production in LPS-stimulated RAW 264.7 cells. In response to LPS stimulation, as expected, cells released more TNF-α (*p* < 0.0001) than control cells (Fig. 1c). However, when LPS-activated cells were pre-treated with increasing concentrations of the extract, there was a dose-dependent decrease in TNF-α level (*p* < 0.001), indicating a possible anti-inflammatory effect of the extract. In addition, we studied the effect of *M. plinioides* extract on protein expression of inflammation-associated markers (NF-kB, p38α and p-p38α MAPK) in LPS-activated RAW 264.7 cells. As shown in Fig. 1d, e, LPS increased p38α phosphorylation, whereas the extract was capable to inhibit LPS-induced phosphorylation. A more significant inhibition (*p* < 0.001) of p38α phosphorylation was seen in cells treated with 50 μg/mL extract, suggesting that this suppression might be involved in the inhibition of LPS-induced TNF-α release in RAW 264.7 cells. We also evaluated the NF-κB (p65) and Fig. 1f shows that p65 protein expression was increased by LPS, while the extract inhibited this stimulation in a dose-dependent way. Altogether, these results suggest that *M. plinioides* extract may hinder LPS-induced NF-κB translocation by decreasing the phosphorylation of p38α, and subsequently reducing the production of TNF-α.

### M. plinioides ethanol leaf extract exerts anti-inflammatory effects in rats

**Fig. 2:**
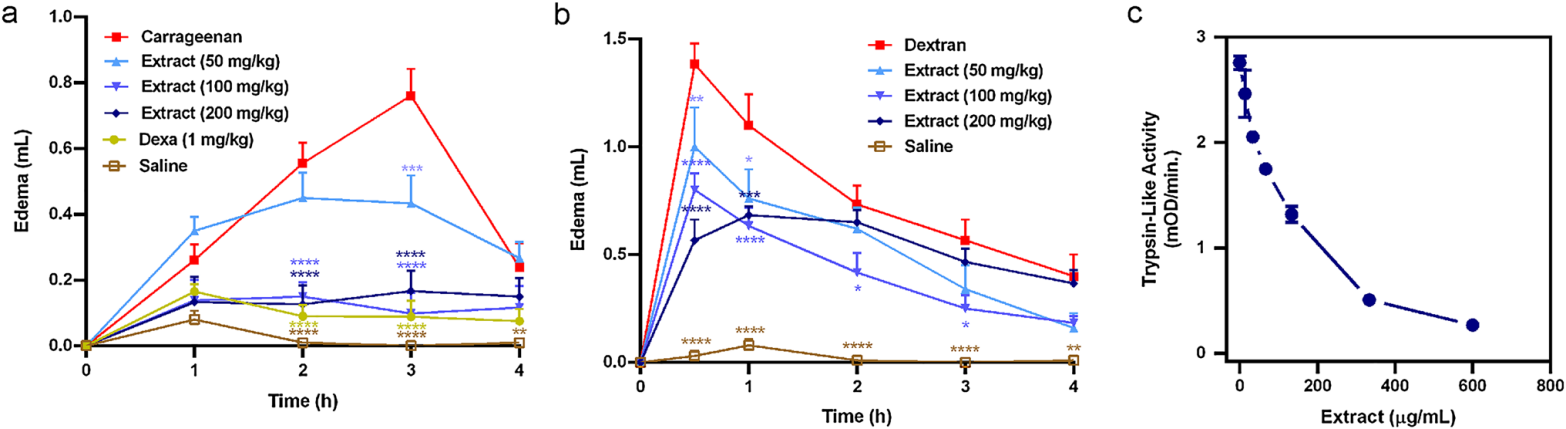
In vivo effects of *M. plinioides* ethanol extract on inflammatory models of rat’s paw edema. **a** Carrageenan-induced paw edema model. Animals were injected with extract (50, 100 and 200 mg/kg; s.c) before carrageenan injection (100 μL; 700 μg/paw; s.c.). Dexamethasone (Dexa) (1 mg/kg; s.c.) was used as a positive control. Two-way ANOVA with Dunnett’s correction for repeated measures. **b **Dextran-induced paw edema model. Animals received extract (50, 100 and 200 mg/kg; s.c.) before dextran injection (100 μL; 500 μg/paw; s.c.). Mean values ± SEM are shown (*n* = 6/group). *p < 0.05, **p < 0.01, ***p < 0.001, ****p < 0.0001 compared with carrageenan or dextran. Two-way ANOVA with Dunnett’s correction for repeated measures. **c** Activity of mast cells proteolytic enzymes. Trypsin-like enzymes of mast cells were extracted and incubated with different concentrations of the extract. The residual amidolytic activity was measured by addition of the BAPNA substrate. Mean values ± SEM are shown (*n* = 3)

To get addition data to confirm the anti-inflammatory potential of *M. plinioides*, we investigated the in vivo effect of the extract using rat paw edema models with two different edema inducers: (i) carrageenan and (ii) dextran. As revealed in Fig. 2a, animals receiving carrageenan injection exhibited intense edema at all time intervals reaching the peak at the third hour. However, animals pre-treated with the extract showed a significant reduction (*p* < 0.001) in carrageenan-induced edema formation. The doses of 100 and 200 mg/kg of extract showed the best results, comparable to corticoid dexamethasone. Figure 2b shows that dextran-induced edema reached a peak in the first 30 min at the time of increased osmotic leakage. This augmented vascular permeability was reduced in the subsequent hours. Pre-treatment with all concentrations of *M. plinioides* extract was shown to be effective in reducing dextran-induced edema (*p* < 0.001), mainly in the first 30 min of administration. The 100 mg/kg extract had the most significant effect during the whole time-course. In addition, the trypsin-like activity of secreted proteases of mast cell granules has also been evaluated. As shown in Fig. 2c, there was an intense inhibition of mast cell secreted trypsin-like enzymes, reaching 90.3% of inhibition in the concentration of 600 µg/mL of extract when compared with control. This inhibition of the trypsin-like enzyme of mast cells provides another important indication of the anti-inflammatory potential of the ethanol extract of *M. plinioides*.

### *Anticoagulant activity of M. plinioides ethanol extract *via* extrinsic pathway*

**Fig. 3:**
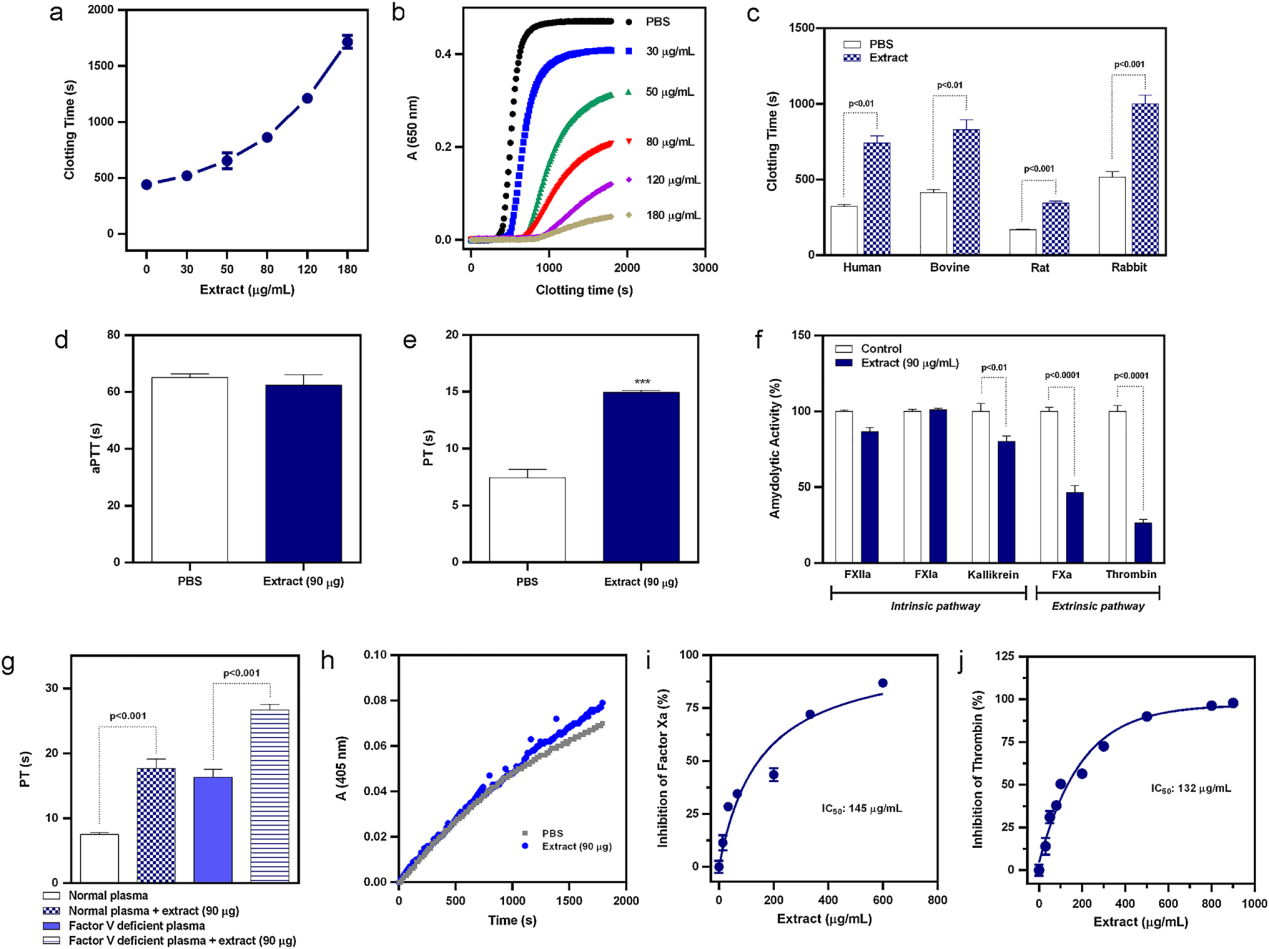
In vitro anticoagulant activity of *M. plinioides* ethanol extract. **a** Recalcification time (RT) inhibition. Increasing concentrations of the extract were incubated with human plasma and coagulation pathway was triggered by the addition of calcium. **b** Representative plot of the dose–response curve of the extract-induced RT inhibition. **c** Anticoagulant activity of 90 μg/mL extract on the RT of different species plasma. **d** and **e** The extract (90 μg/mL) was incubated with normal human plasma and the activated partial thromboplastin time (aPTT) was used to measure the activity of the intrinsic coagulation pathway and the prothrombin time (PT) was used to evaluate the extrinsic pathway, respectively. **f** Inhibitory activity of 90 μg/mL extract on specific factors of the intrinsic and extrinsic pathways of coagulation was measured directly in plasma activated with ellagic acid or tissue factor. **g** Effect of 90 μg/mL extract on factor Va activity. The extract was incubated with normal, or factor V deficient human plasma and PT was recorded after the activation of the extrinsic pathway with thromboplastin. **h** Effect of 90 μg/mL extract on factor VIIa. Factor VII deficient plasma was activated with tissue factor and incubated with the extract. The amidolytic activity of factor VIIa was measured by addition of the substrate SCP-0248. (i) Effect of the extract on factor Xa. Purified factor Xa was incubated with different concentrations of the extract and the residual activity of factor Xa was measured indirectly by the activation of prothrombin. (j) Effect of the extract on thrombin. Purified thrombin was incubated with different concentrations of the extract and the residual activity of the enzyme was monitored by fibrin formation. Mean values ± SEM are shown (*n* = 3). ***p < 0.001 compared with PBS

To explore the mechanisms underlying the anticoagulant potential of *M. plinioides*, the effects of several concentrations of the extract were analysed on the coagulation pathway. First, we investigated the recalcification time (RT). Increasing concentrations (30, 50, 80, 120 and 180 μg/mL) of the extract were incubated with human plasma and the common coagulation pathway was triggered by the addition of calcium. According to Fig. 3a, all concentrations of the extract extended the beginning of coagulation process. This delay in the coagulation cascade was more prominent with the highest concentration (180 μg/mL) of the extract, which extended the onset of clotting about 3.8 times over the clotting time of extract-free plasma. Analyzing the kinetic profile of the process (Fig. 3b), it was also possible to observe that the anticoagulant activity of the extract was mainly due to the inhibition of the enzymatic activity of coagulation factors and not due to its fibrinolytic action. There was an evident reduction of the maximum optical density of the reactions as well as a prolongation of the initial phase (lag phase) of the coagulation cascade.

The activity of the extract was subsequently assessed on human, bovine, rat and rabbit plasmas. As shown in Fig. 3c, the extract exerted effective anticoagulant activity in all evaluated plasmas as it prolonged the clotting time when compared with PBS (control). The fact that the extract exhibited anticoagulant activity in all different plasmas demonstrates that the inhibitor(s) contained in the extract are not species specific as it blocked enzymes of different species regardless of their structural differences.

Furthermore, the effects of the extract were evaluated on clot formation, which can be triggered by two distinct pathways, extrinsic and intrinsic. Figure 3d shows that the aPTT was not modified by the extract. However, the PT was prolonged by the extract up to 1.9 times (*p* < 0.001; Fig. 3e), indicating *M. plinioides* extract may act preferentially by the extrinsic pathway. The inhibitory effect of the extract on the amidolytic activity of coagulation factors FXIIa, FXIa and kallikrein (intrinsic pathway), and FXa and thrombin (extrinsic pathway) was measured directly in ellagic acid or tissue factor-activated human plasma. According to Fig. 3f, there was a slight inhibition (19.9%) of the intrinsic factor kallikrein. Meanwhile, a marked inhibition was seen on the activity of the extrinsic factors. The extract was able to inhibit 53.4% of FXa activity and 73.6% of thrombin activity, suggesting that the extract acts extrinsically, mainly through thrombin inhibition.

Table 1 summarize the anticoagulant potential of some plant extracts and isolated compounds that demonstrated inhibitory activity on thrombin in our experimental trials. When comparing the IC_50_ of *M. plinioides* extract with the IC_50_ of other extracts, we can highlight that *M. plinioides* exhibits the most potent inhibitory activity on thrombin. In addition, when comparing it with isolated compounds, the extract also demonstrated a great potential, achieving the second best IC_50_.

**Table 1:** Comparison of different plant extracts or isolated natural compounds with inhibitory activity on thrombin

Natural product	Extract/Compound	IC_50_^a^
*Myrciaria plinioides*	Ethanol extract	132 ± 8 μg/mL
*Ilex paraguariensis* A. St. Hil	Aqueous extract	146 ± 8.9 μg/mL
*Thinouia coriacea* Britton	Aqueous extract	1012 ± 51 μg/mL
Glicirrizine	Triterpenoid saponin	182.5 ± 11 μg/mL
Chikusetsusaponin	Triterpenoid saponin	115 ± 6 μg/mL
Ursolic acid	Triterpene	391 ± 26 μg/mL

^a^IC_50_ values represent the concentration of extract or isolated compound capable of reducing the catalytic activity of thrombin by 50% on fibrinogen. Data represent the mean ± standard error (*n* = 3)

Then, further investigation o were done to elucidate the mechanism of action of *M. plinioides* extract on the extrinsic pathway. The activity of key extrinsic factors (FV, FVII, FX and thrombin) were examined. Figure 3g depicts the activity of FVa in normal human plasma and FV deficient plasma after the activation of the extrinsic pathway with thromboplastin. The extract prolonged the PT of FV deficient plasma, which discards the possibility of a specific inhibitor of FVa. Similarly, the presence of a specific inhibitor of FVIIa was also discarded (Fig. 3h). In contrast to what was observed for FVa and FVIIa, the extract exhibited a potent and direct inhibitory effect on FXa and thrombin (Fig. 3i, j, respectively). When assessed in an isolated system (using both purified FXa and thrombin), the extract dose-dependently inhibited the activity of these enzymes on their macromolecular substrates, prothrombin and fibrinogen, respectively. The hampering of thrombin activity (Fig. 3j) plus the results of Fig. 3f emphasize the anticoagulant potential of the extract via the extrinsic pathway.

## Discussion

Borges et al. (2014) provided an overview of the active compounds present in several species of the genus *Myrciaria.* Although *M. plinioides* was not included in their study, the data of other *Myrciaria* species corroborate with our previous analyses that did not show coumarins and quinones as phytoconstituents. Previously, our group performed a phytochemical analysis of the fingerprints of *M. plinioides* EtOH extract and it was detected seven compounds including: caffeic acid, catechin, *p*-coumaric acid, rutin, quercetin, luteolin and cyanidin chloride (Marmitt et al. 2019). These polyphenols are usually related to the biological potential of plant extracts, mainly due to their known antioxidant effects (Marchelak et al. 2017). In addition, the major structural groups of the EtOH extract were characterized by nuclear magnetic resonance (NMR) spectroscopy (data not published). The detected peaks suggest the presence of terpenoid compounds, which were also identified in other *Myrciaria* species (Table 2), such as *Myrciaria peruviana* (Poir.) Mattos (Ishikawa et al. 2008). Sugar components peaks were also detected, which may be attached to phenolic structures. In addition, some phenolic acids such as caffeic acid and *p*-coumaric acid were detected (Marmitt et al. 2019).

**Table 2:** Main compounds identified in *Myrciaria* genus

Plant specie	Compound(s)	Reference
*Myrciaria cauliflora (*Mart.) O. Berg	Cyanidin-3-*O*-glucoside; delphinidin-3-*O*-glucoside; quercitrin; syringin; succinic acid; oxalic acid; tartaric acid	Wu et al. 2012
	Ascorbic acid	Reynertson et al. 2008
	Gallic acid; protocatechuic acid; kaempferol; epi-gallocatechin-3-O-gallate; quercetin	Hussein et al. 2003
	Quercimeritrin; quercitrin; rutin; myricitrin; pyranocyanins; cinnamic acid; *O*-coumaric acid; gallic acid; methyl protocatechuate	Einbond et al. 2004
	Protocatechuic acid	Hussein et al. 2003/Einbond et al. 2004
	Isoquercitrin; myricitrin; ellagic acid	Wu et al. 2012/Einbond et al. 2004
*Myrciaria dubia* (Kunth) McVaugh	Catechin; flavan-3-ol; rutin; cyanidin-3-glucoside; delphinidin 3-glucoside	Reynertson et al. 2008
	Ellagic acid; 4-O-methylellagic acid; 4-(α-rhamnopyranosyl) ellagic acid	Akter et al. 2011
	α-pinene; d-limonene; β-caryophyllene	Franco and Shibamoto 2000
*Myrciaria plinioides* D. Legrand	Caffeic acid, catechin, *p*-coumaric acid, rutin, quercetin, luteolin and cyanidin chloride	Marmitt et al. 2019
*Myrciaria* *vexator* McVaugh	Cyanidin-3-O-glucoside; delphinidin-3-O-glucoside; jaboticabin; 2-O-(3,4-dihydroxybenzoyl)-2,4,6-trihydroxyphenylacetic acid; ellagic acid; quercitrin; rutin; myricitrin; protocatechuic acid; methyl protocatechuate; cyanidin galactoside	Dastmalchi et al. 2012
*Myrciaria floribunda* (H. West ex Willd.) O. Berg	2E, 6E-farnesyl acetate; 1,8-cineole	Tietbohl et al. 2012
*Myrciaria trunciflora* O.Berg	Bicyclogermacrene; γ-muurolene; β-caryophyllene; globulol;	Apel et al. 2006
*Myrciaria edulis* (Vell.) Skeels	β-caryophyllene; caryophyllene oxide	Apel et al. 2006
*Myrciaria peruviana* (Poir.) Mattos	β-caryophyllene	Ishikawa et al. 2008
*Myrciaria cordifolia* D. Legrand	α-bisabolol oxide A	Apel et al. 2006

Several biological and pharmacological activities were described for *Myrciaria* species, including antioxidant and anti-inflammatory **(**Borges et al. 2014; Naspolini et al. 2016). These effects were demonstrated by our group in a previous study, showing a potent antioxidant activity and a significant amount of the total phenolic content (65.07 mg GAE/g extract) (Marmitt et al. 2019). In addition, polyphenol-rich extracts have potential as alternative ingredients in the preparation of pharmaceutical drugs for a wide range of pathologies, including inflammatory diseases (Newman and Cragg 2020).

Beyond the potent antioxidant activity, the extract of *M. plinioides* has significant neuroprotective activity, which occurs due to the antioxidant effects, preventing the depolarization of the membrane and significantly decreasing the production of H_2_O_2_ and the activity of caspase-3 (Marmitt et al. 2019). These activities can be attributed to the identified phenolic compounds, such as caffeic acid and quercetin known for their antioxidant and anti-inflammatory properties (Lee et al. 2018; Chao et al. 2009), results that corroborate to explore *M. plinioides* anti-inflammatory activity. Moreover, macrophages play a crucial role during inflammatory response due to secrete a variety of factors including inflammatory cytokines, such as TNF-α, while the endotoxin LPS is one of the major activators of macrophages (Murray and Wynn 2011) and a potent activator of MAPK pathway (Lloberas et al. 2016). Furthermore, extracellular regulation of p38 MAPK is considered a key element in the intracellular signaling cascades responsible for the activation of NF-κB and its dysregulation has been associated with oxidative stress and inflammatory diseases (Surh et al. 2001). In addition, we suggested that ethanol extract presented a strong in vitro inhibitory activity toward p38 MAPKs and also toward TNF-α release in human whole blood (Marmitt et al. 2019).

The in vivo results were crucial to confirm our hypothesis that *M. plinioides* extract was anti-inflammatory. The extract not only decreased the edema in both models, as it reached the same response as the standard anti-inflammatory drug. In the first model, it was used the phlogistic agent carrageenan, which induces intense cellular edema by means of a completely localized inflammatory response in rodents (Di Rosa and Sorrentino 1968). The formation of edema is via induction of acute local inflammation and involves the release of several mediators, cell migration and plasma exudation (Loram et al. 2007). Thus, animals receiving carrageenan injection reached the edema peak at the third hour, which is the moment of greatest cellular infiltration (Hajare et al. 2001). The second edema model used the common osmotic edema inducer dextran. As previously reported, dextran induces local irritation via activation of kinins and release of vasoactive amines, such as histamine and serotonin, leading to degranulation of mast cells (De Araújo et al. 2016).

**Fig. 4:**
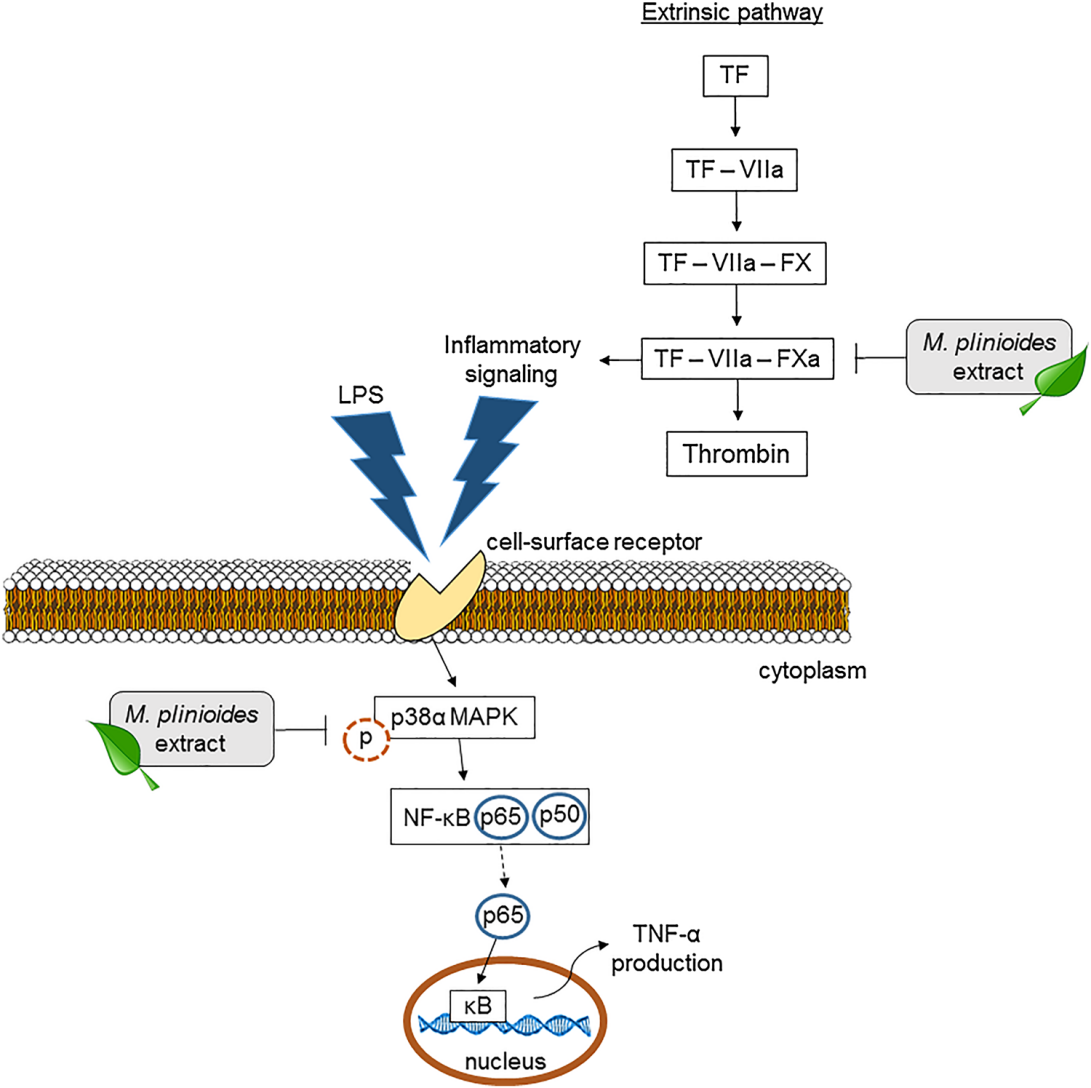
Scheme of the possible anticoagulant and anti-inflammatory mechanisms of *M. plinioides* ethanol extract. *M. plinioides* extract blocks thrombin activity via the extrinsic pathway. In addition, the extract acts in the cytosol hampering LPS-induced NF-κB translocation by decreasing the phosphorylation of p38α, and subsequently reducing the production of TNF-α. FX, factor X; TF, tissue factor; VIIa, factor VIIa

The proposed mechanism of action of *M. plinioides* ethanol leaf extract is described in Fig. 4. Perhaps, the effects of *M. plinioides* are due to inhibitors contained in the extract that may be acting as inhibitors of clotting factors and as anti-inflammatory agents. These detected metabolites have known anti-inflammatory and anticoagulant activities. For example, the flavonoids quercetin, rutin and luteolin have similar suppressive effects on the carrageenan-induced inflammation (Siebert et al. 2017). They modulate the prostanoid synthesis as well as cytokines production (e.g., TNF-α) (Morikawa et al. 2003). Catechin inhibits inflammation through antioxidant activity. Also, it can regulate infiltration and proliferation of immune related-cells, such as neutrophils, macrophages, and T lymphocytes leading to a decrease of inflammatory reactions (Fan et al. 2017). Caffeic acid has multiple biological effects, including antioxidant and anti-inflammatory effects. It modulates inflammatory cytokines (TNF-α, IL-6 and IL-1β) via inhibition of I-κB-α degradation and p65 phosphorylation in the NF-κB pathway besides suppressing the activity of pathways, such as the MAPKs (Liu et al. 2014). In addition, *p*-coumaric acid has in vivo and in vitro antiplatelet activity. Its effects can be obtained by dietary doses, suggesting a possible application for primary prevention of vascular disease (Luceri et al. 2007).

The main pathway that link inflammation and hemostasis is the TF-FVII-PAR pathway (Pawlinski et al. 2004). Once activated this pathway can generate the main procoagulant enzymes, such as FXa and thrombin, which can, in turn, cleave PAR receptors on platelets and endothelial cells amplifying the proinflammatory signal (Landis 2007). One of the most relevant find of this work is that *M. plinioides* ethanol leaf extract can inhibit efficiently proinflammatory proteolytic enzymes secreted by mast cells and also the main procoagulant molecules, such as thrombin and FXa. In fact, the inhibitory potential of *M. plinioides* ethanol leaf extract on proteases is an important mechanism of action observed herein, since proteolytic enzymes have key functions not only in the paw edema in vivo assay but also in the coagulation assays. *M. plinioides* ethanol leaf extract was able to block specifically the extrinsic pathway of blood coagulation cascade without any effect on intrinsic pathway. Although the extract did not directly inhibit FVIIa, it was able to inhibit FXa and thrombin, which are the main products of the extrinsic cascade. For this reason, the extract had an efficiently anticoagulant effect. Until now, the main class of metabolites described in natural products able to block procoagulant enzymes are saponins and flavonoids (Bijak et al. 2014a). Our group was the first to described two glycosylated triterpenoid saponins, glycyrrhizin and chickusetsusaponin IV, as specific thrombin inhibitors (Francischetti et al. 1997; Mendes-Silva et al. 2003; Dahmer et al. 2012). Both molecules can bind and inhibit thrombin via the anion binding exosite 1, blocking the interaction of the enzyme with its main physiological substrate, fibrinogen (De Paula et al. 2013). Similarly, also natural flavonoids and sulphated flavonoids have been described as efficient anticoagulant and antiplatelet agents (Guglielmone et al. 2005; Mozzicafreddo et al. 2006; Bijak et al. 2014b). Flavonoids such as quercetin, rutin, luteolin and hyperosid have antithrombin activity, both in vitro and in vivo (Mozzicafreddo et al. 2006). Between these molecules quercetin, had the lower Kd (~ 0.23 µM) and docking studies indicated that its mechanism of thrombin inhibition was related to the planar structure of quercetin which is able to directly interact via hydrogen bond to His57, one of the key amino acids in the thrombin catalytic site (Mozzicafreddo et al. 2006). Therefore, we can speculate that these flavonoids could be involved in the biological effects observed here. Despite these flavonoids are efficient inhibiting procoagulant enzymes, little is known about its ability to block proinflammatory enzymes, such as those found in mast cells. Actually, we are focused on to identify the molecules responsible for this effect.

## Conclusion

Our results demonstrate that *M. plinioides* has great biological potential in the form of the investigated extract. Considering that the antioxidant and neuroprotective effects have been previously described, the anti-inflammatory activity in vivo, the inhibitory effect on pro-inflammatory enzymes and the inhibitory effect of the extrinsic pathway of clot formation, suggest that the ethanolic extract of *M. plinioides* may be a promising candidate for the development of anti-inflammatory and anticoagulant drugs. Yet, its potential as a food cannot be discarded (Fig. 4). Our future study will focus on the isolation and elucidation of the active secondary metabolites responsible for the respective biological activities.

## Data Availability

Not applicable.
